# The complete mitogenome of the Atlantic longnose chimaera *Rhinochimaera atlantica* (Holt & Byrne, 1909)

**DOI:** 10.1080/23802359.2024.2378127

**Published:** 2024-07-17

**Authors:** Ana Matos, Nair Vilas-Arrondo, André Gomes-dos-Santos, Ana Veríssimo, Esther Román-Marcote, Francisco Baldó, Jaime Moreno-Aguilar, Montse Pérez, Manuel Lopes-Lima, Elsa Froufe, L. Filipe C. Castro

**Affiliations:** a CIIMAR/CIMAR – Interdisciplinary Centre of Marine and Environmental Research, University of Porto, Matosinhos, Portugal; b Programa de Doctorado “Ciencias marinas, Tecnología y Gestión” (Do*MAR), Universidad de Vigo, Vigo, Spain; c Centro Oceanográfico de Vigo (COV), Instituto Español de Oceanografía (IEO), CSIC, Vigo, Spain; d CIBIO, Centro de Investigação em Biodiversidade e Recursos Genéticos, InBIO Laboratório Associado, Universidade do Porto, Vairão, Portugal; e BIOPOLIS Program in Genomics, Biodiversity and Land Planning, CIBIO, Vairão, Portugal; f Centro Oceanográfico de Cádiz (COCAD), Instituto Español de Oceanografía (IEO), CSIC, Cádiz, Spain; g Tecnologías y Servicios Agrarios, S.A. (TRAGSATEC), C/ Orient, Ciutadella, Spain; h Department of Biology, Faculty of Sciences, University of Porto, Porto, Portugal

**Keywords:** Holocephali, mitochondrial genome, chondrichthyans

## Abstract

Holocephali is a subclass of chondrichthyans with ample geographic distribution in marine ecosystems. Holocephalan species are organized into three families: Callorhinchidae, Chimaeridae, and Rhinochimaeridae. Despite the critical ecological and evolutionary importance, genomic information from holocephalans is still scarce, particularly from rhinochimaerids. The present study provides the first complete mitogenome of the Atlantic longnose chimaera *Rhinochimaera atlantica* (Holt & Byrne, 1909). The whole mitogenome was sequenced from an *R. atlantica* specimen, collected on the Porcupine Bank (NE Atlantic), by Illumina high-throughput sequencing. The *R. atlantica* mitogenome has 17,852 nucleotides with 13 protein-coding genes, 22 transfer RNA, and two ribosomal RNA genes. Nine of these genes are in the complementary strand. This mitogenome has a GC content of 41.5% and an AT content of 58.5%. The phylogenetic reconstruction provided here, using all the available complete and partial Holocephali mitogenomes, places *R. atlantica* in the Rhinochimaeridae family, as expected. This genomic resource will be useful in the genomic characterization of this species.

## Introduction

Holocephali comprises mostly deep-water species widely distributed, except for the Antarctic ecosystem (Didier et al. 2012). This subclass of Chondrichthyes comprises nearly 40 species and only one taxonomic order (Chimaeriformes) with three families (Callorhinchidae, Chimaeridae, and Rhinochimaeridae) (Compagno et al. 2005; Weigmann 2016). Rhinochimaeridae specimens are characterized by a conspicuously long and flat snout, large head, and elongated body (Carpenter 2002; Didier et al. 2012) and inhabiting water depths between 400 m and more than 2000 m (Carpenter 2002; Weigmann 2016). Currently, this family comprises three genera (*Harriotta*, *Neoharriotta*, and *Rhinochimaera*) (Finucci et al. 2017). For the three *Rhinochimaera* species, two complete mitogenomes of *Rhinochimaera pacifica* and one partial mitogenome of *Rhinochimaera africana* are available (accessed on 1 December 2023). However, a complete mitogenome of *Rhinochimaera atlantica* (Holt & Byrne, 1909) (Atlantic longnose chimaera) is not available (accessed on 1 December 2023). The presence of *R. atlantica* has been documented in South Africa, the North Atlantic Ocean, the Gulf of Mexico (WORMS, accessed on 1 December 2023) (Carpenter 1969), and the western Indian Ocean (Weigmann 2016) (Supplementary Figure S1). *Rhinochimaera atlantica* is listed as ‘Least Concern’ by the International Union for Conservation of Nature (IUCN) (Finucci 2020). Yet, its biological characteristics as slow growth, low fecundity, or the tendency to aggregate contribute to its overfishing and consequent vulnerability (Stevens et al. 2000; Barnett et al. 2012; Finucci et al. 2017). The biological and evolutionary importance of Chondrichthyes has boosted the development of studies aiming to provide genomic resources helpful in the study of these species (e.g. Vilas-Arrondo et al. 2022; Yamaguchi et al. 2023). However, there is a substantial lack of molecular data which, in combination with morphological characters, is crucial for describing new Chimaeridae species and elucidating relationships among extant holocephalans (Licht et al. 2012; Iglésias et al. 2022). Hence, the present study provides the first complete mitogenome of *R. atlantica*.

## Materials and methods

**Figure 1.**
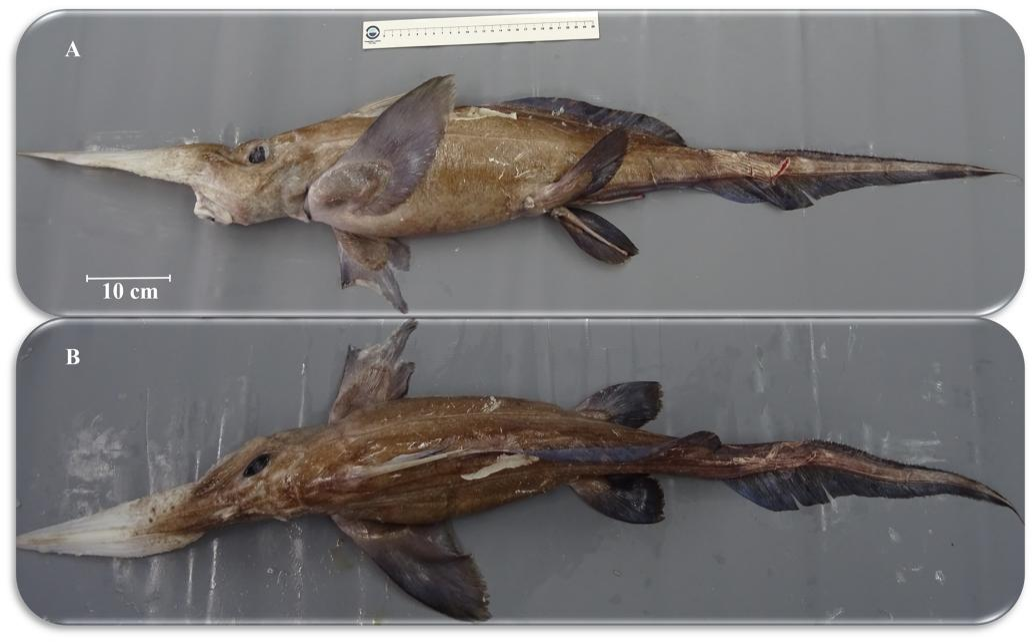
Species reference image of *Rhinochimaera atlantica.* The most characteristic feature is its faction rhinoceros’ nose, in reference to the long and pointed proboscis. Photograph by Francisco Baldó onboard the R/V Vizconde de Eza during the Spanish Bottom Trawl Survey on the Porcupine Bank.

On 28 September 2021, a male specimen of *R. atlantica* was collected in the Porcupine Bank (51.301200, −13.211200)), North-East Atlantic, at a depth of 1435 m (Supplementary Figure S1) during the Spanish Bottom Trawl Survey on the Porcupine Bank. Sampling was carried out onboard the R/V Vizconde de Eza, a stern trawler of 53 m and 1800 kW, using a Baca-GAV 39/52 with a cod-end mesh size of 20 mm. The specimen (voucher name k141_3), which was morphologically identified on board, had a Pre Supra Caudal Fin Length (PSCFL) of 814 mm and weighed 3050 g (Figure 1). The initial identification of the specimen took place on board during the scientific survey conducted by experienced researchers specializing in the species from the Porcupine Bank, including Francisco Baldó. To achieve this, they gathered data on the total length, weight, and sex of the individual, with particular emphasis on the intrinsic and distinctive characteristics of the chimaera as outlined in Ebert and Dando (2020). These features comprise a very long strait conical snout, an underside of snout whitish, a mouth well in front of the eyes, a lateral head profile straight, eyes very small, a margin of upper caudal fin lobe with tubercules, a height of the upper caudal fin lobe less than height of lower caudal fin lobe and filamentous tail short. The pectoral fins are narrow, and the caudal fin exhibits an exceptionally short terminal filament and widely spaced tubercles along the upper edge in adults and large juveniles (Compagno et al. 1989; Ebert and Dando 2020). The coloration of this species ranges from whitish to light brown (Compagno et al. 1989). Additionally, upon reaching land, the specimen was sent to the laboratory of the Spanish Institute of Oceanography COV-CISC. There, the identification of the chimaera was confirmed by experienced personnel in Chondrichthyes, namely Nair Vilas Arrondo and Esther Roman Marcote. A total of 92 morphometric measurements were conducted following the protocols outlined by James et al. (2009), Compagno (1990), and Didier and Nakaya (1999), as part of the framework of Nair’s doctoral thesis, which complements the taxonomic identification (Supplementary Table S1). The specimen was deposited at the Oceanographic Center of Vigo, IEO-CSIC (https://www.ieo.es/es/web/vigo/, Nair Vilas Arrondo, nair_vilasarrondo@hotmail.com) with voucher name k141_3.

Total genomic DNA was extracted using a standard high-salt protocol (Sambrook et al. 1989) and sent to Macrogen (Seoul, South Korea) for Illumina Paired-End (PE) library construction (2 × 150 bp) and whole genome sequencing on a Novaseq6000 platform. Adapters, in the raw PE sequencing reads, were removed with Trimmomatic (version 0.38) by setting parameters leading and trailing to 5, minlen of 36, and a ‘sliding window’ of 4 bp with the required quality of 15 (Bolger et al. 2014). Mitogenome assembly and annotation were conducted with MitoZ (version 3.4) (Meng et al. 2019) with the clean PE reads and default parameters, using the following associated tools: GeneWise (Birney et al. 2004), Blast+ (Gertz et al. 2006), Cricos (Krzywinski et al. 2009), BWA (Li and Durbin 2009), SAMtools (Li et al. 2009), MiTFi (Jühling et al. 2012), Infernal (Nawrocki and Eddy 2013), HMMER (Wheeler and Eddy 2013), Megahit (Li et al. 2015), ete3 (Huerta-Cepas et al. 2016), Fastp (Chen et al. 2018), and tiara (Karlicki et al. 2022). The mitochondrial reads were retrieved from the whole genome sequencing outputs, by mapping the trimmed reads to the mitogenome assembly using the bbmap v.37.77 and after selecting only the mapped reads using the tool reformat.sh (implemented through bbmap) (Bushnell 2014). The mitogenome map was created with the annotation module of MitoZ. To generate a coverage plot, the PE reads were mapped to the final assembly using Burrows–Wheeler Aligner v.0.7.17-r1198 (Li 2013) and after bam2plot (https://github.com/willros/bam2plot) was used to generate the graphical plot (Supplementary Figure S2). All the available complete and partial Holocephali mitogenome assemblies (with more than 10,000 bp) were downloaded (*n* = 16) from GenBank (accessed on 1 December 2023). Two complete elasmobranch mitogenomes (one representing Rajiformes and one representing Squalomorphii) were also downloaded from GenBank and used as outgroup taxa.

The alignment of the 13 protein-coding genes (PCGs) retrieved from the downloaded mitogenomes was conducted with MAFFT (version 7.505) (Katoh and Standley 2013), trimmed with trimAL (version 1.2) (Capella-Gutiérrez et al. 2009) and concatenated with FasConCAT-G (version 1.05.1) (Kück and Longo 2014). The final alignment had a total of 11,418 bp. The identification of the partition-scheme, best-fit nucleotide substitution models, and maximum-likelihood phylogenetic inference (10,000 ultrafast bootstrap replicates) were conducted on IQ-TREE (version 1.6.12) (Nguyen et al. 2015; Kalyaanamoorthy et al. 2017).

## Results

**Figure 2.**
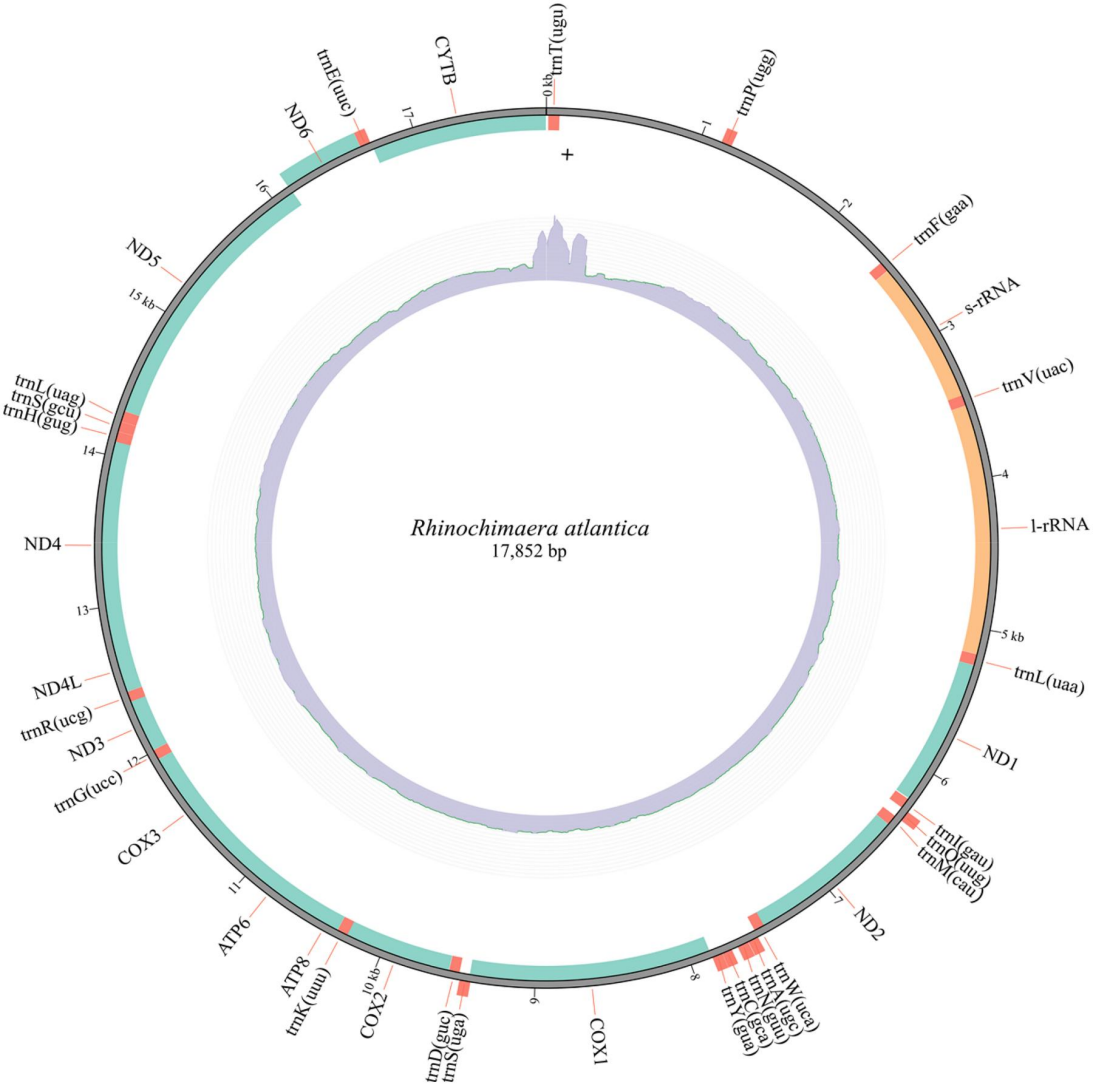
Mitogenome map of *Rhinochimaera atlantica* with 17,852 bp, 13 protein-coding genes (*ND1*, *ND2*, *COX1*, *COX2*, *ATP8*, *ATP6*, *COX3*, *ND3*, *ND4L*, *ND4*, *ND5*, *ND6*, and *CYTB*), 22 transfer RNA (beginning by ‘trn’), and two ribosomal RNA genes (s-rRNA and l-rRNA). Nine of these genes (one protein-coding gene (*ND6*) and eight transfer RNA – *trnP(ugg)*, *trnQ(uug)*, *trnA(ugc)*, *trnN(guu)*, *trnC(gca)*, *trnY(gua)*, *trnS(uga)*, and *trnE(uuc)*) are in the complementary strand, demonstrated in the outside of the circle. The inner circle represents the coverage plot (blue).

**Figure 3.**
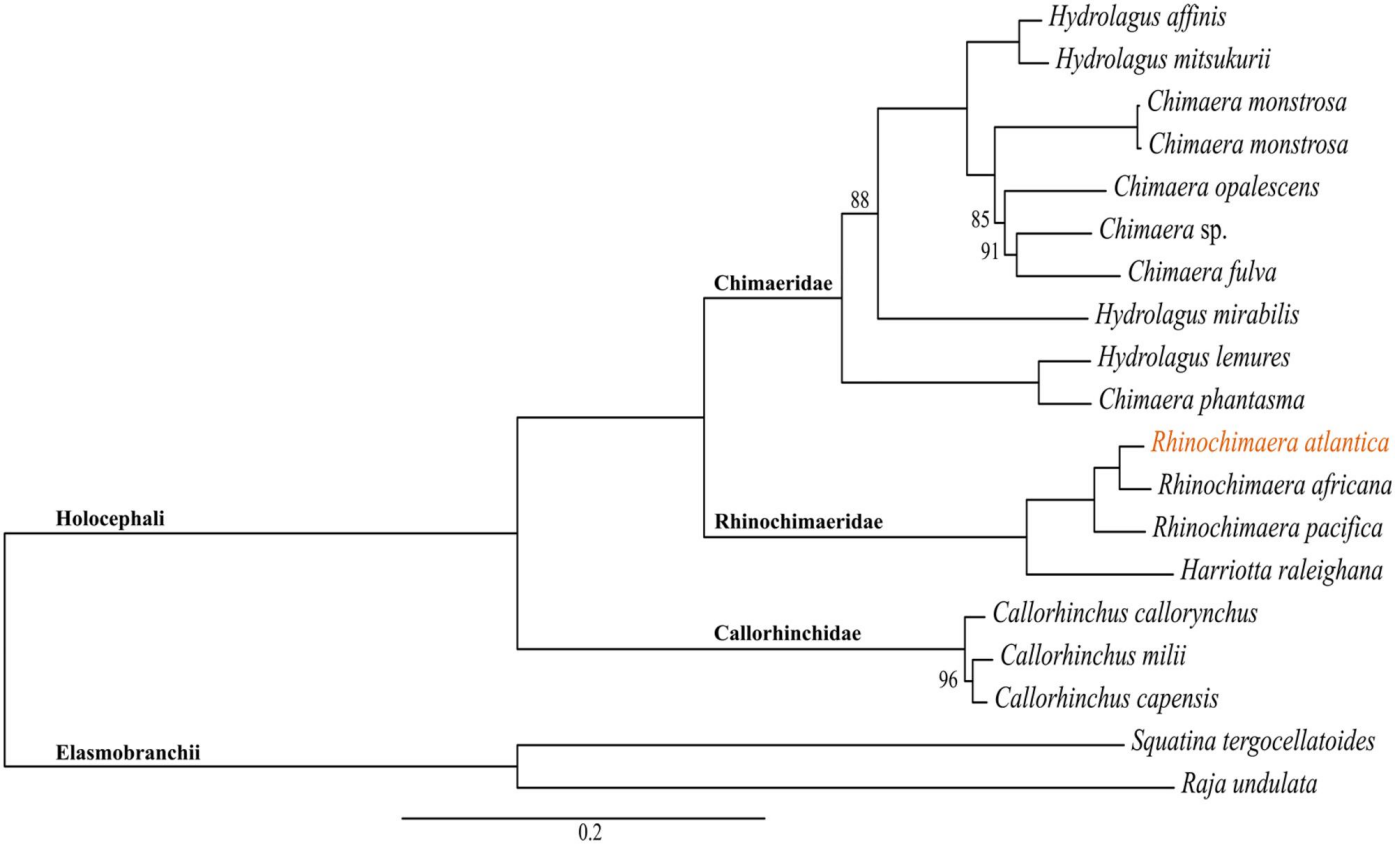
Maximum-likelihood phylogenetic inference with the sequences of all protein-coding genes from all available Holocephali mitogenomes (*n* = 17). The sequences were downloaded from GenBank (accessed on 1 December 2023) with the following accession numbers: MT090368 (*H. affinis*) (Gomes-dos-Santos et al. 2020), OP391486 (*H. mitsukurii*) (Wang et al. 2023), MT410927 (*C. monstrosa*) (unpublished), AJ310140 (*C. monstrosa*) (Arnason et al. 2001), OK638184 (*C. opalescens*) (Vilas-Arrondo et al. 2022), MT880605 (*Chimaera* sp.) (unpublished), HM147138 (*C. fulva*) (Inoue et al. 2010), MW029477 (*H. mirabilis*) (Gomes-dos-Santos et al. 2021), HM147139 (*H. lemures*) (Inoue et al. 2010), KP006329 (*C. phantasma*) (unpublished), KP006330 (*R. africana*) (unpublished), HM147141 (*R. pacifica*) (Inoue et al. 2010), HM147140 (*H. raleighana*) (Inoue et al. 2010), HM147135 (*C. callorhynchus*) (Inoue et al. 2010), HM147137 (*C. milii*) (Inoue et al. 2010), and HM147136 (*C. capensis*) (Inoue et al. 2010). Outgroup taxa: ON210850 (*S. tergocellatoides*) (unpublished) and MT274574 (*R. undulata*) (Kousteni et al. 2021). The mitogenome of *R. atlantica* provided in this study was also added to this phylogenetic inference (accession number: OQ947382). Only the bootstrap support values lower than 100 are represented in the figure.

The assembled mitogenome of *R. atlantica* with 17,852 bp has 13 PCGs, 22 transfer RNA, and two ribosomal RNA genes (Figure 2). Nine of these genes (one PCG, NADH dehydrogenase subunit 6 (*ND6*), and eight transfer RNA – *trnP(ugg)*, *trnQ(uug)*, *trnA(ugc)*, *trnN(guu)*, *trnC(gca)*, *trnY(gua)*, *trnS(uga)*, and *trnE(uuc)*) are in the complementary strand. All PCGs started with the ATG codon, except for the cytochrome c oxidase subunit 1 (*COX1*), which started with GTG. The nucleotide composition of the *R. atlantica* mitogenome is A = 30%, T = 28.5%, G = 14.3%, and C = 27.2%, with a GC content of 41.5%. Overlap of sequences was detected in eight mitochondrial genes, ranging between 1 and 13 bp. The mitogenome has been deposited in GenBank under accession number OQ947382. The phylogenetic inference (Figure 3) demonstrates the three Holocephali families to be monophyletic, and that Chimaeridae and Rhinochimaeridae are sister groups with strong support (100% bootstrap probabilities). *R. atlantica* is placed in the Rhinochimaeridae clade.

## Discussion and conclusions

The length of the *R. atlantica* mitogenome is within the range of the two available *Rhinochimaera* mitogenomes (one from *R. pacifica* (24,889 bp) and one from *R. africana* (15,446 bp)) (Inoue et al. 2010). A long noncoding region (between *trnT(ugu)* and *trnP(ugg)* genes) with variable size has been described in holocephalans mitogenomes and pointed as the main contributor to the size variation observed in these mitogenomes (Inoue et al. 2010). In the *R. atlantica* mitogenome sequence provided here, this region is present with a size of 1086 bp. Moreover, this well-reported mitochondrial feature offers challenges to the genome assembler thus explaining the positive coverage variation observed (Supplementary Figure S2). In vertebrates, the control region (CR) is located within the tRNA-Phe and tRNA-Pro (Satoh et al. 2016). In our assembly, this region is well resolved, it has 1222 bp (Figure 2) and has a similar size to other mitogenome assemblies from the same family Rhinochimaeridae (e.g. 1304 bp in *Rhinochimaera pacifica* – HM147141.1; 1316 bp in *Harriotta raleighana* – HM147140.1).

In the phylogeny provided here (Figure 3), *R. atlantica* is in the Rhinochimaeridae clade, as expected. This clade has a group, recovered with 100% bootstraps probabilities, with all *Rhinochimaera* species (including our *R. atlantica* mitogenome), and *Harriotta raleighana* as its sister species (Figure 3). Rhinochimaeridae encompasses three genera, *Rhinochimaera*, *Harriotta*, and *Neoharriotta*, with the latter suggested to be the most basal of these genera (Licht et al. 2012). However, in our phylogenetic analysis, only the mitogenomes of *Rhinochimaera* and *Harriotta* were considered as no *Neoharriotta* mitogenomes are presently available. Comparatively, Chimaeridae in this phylogeny has a wider taxonomic representation (Figure 3).

We provide the first complete *R. atlantica* mitogenome. Among Chondrichthyes, there is a lack of molecular data for Holocephali. This mitogenome will contribute to the genomic characterization of *R. atlantica*. The need to produce more complete Holocephali mitogenomes is highlighted.

## Supplementary Material

Supplemental Material

Supplemental Material

Supplemental Material

## Data Availability

The genome sequence data that support the findings of this study are openly available in GenBank of NCBI at https://www.ncbi.nlm.nih.gov under the accession number OQ947382. The associated BioProject, SRA, and Bio-Sample numbers are PRJNA1055343, SRR27310001, and SAMN38990799, respectively.
