# Supplementary material for: The complete mitogenome of the Atlantic longnose chimaera Rhinochimaera atlantica (Holt & Byrne, 1909)
Source: Mitochondrial DNA B Resour. 2024 Jul 17;9(7):886–91. doi: 10.1080/23802359.2024.2378127 (PMC11257016; doi:10.1080/23802359.2024.2378127)
Supplement: Supplemental Material [file TMDN_A_2378127_SM0330.docx]

**Supplementary Material**

The complete mitogenome of the Atlantic longnose chimaera *Rhinochimaera atlantica* (Holt & Byrne, 1909)

Ana Matos^1,+^, Nair Vilas-Arrondo^2,3,*+^, André Gomes-dos-Santos^1^, Ana Veríssimo^4,5^, Esther Román-Marcote^3^, Francisco Baldó^6^, Jaime Moreno-Aguilar^7^, Montse Pérez^3^, Manuel Lopes-Lima^4,5^, Elsa Froufe^1,^* and L. Filipe C. Castro^1,8,^*

^1^CIIMAR/CIMAR - Interdisciplinary Centre of Marine and Environmental Research, University of Porto, Matosinhos, Portugal;

^2^Programa de Doctorado “Ciencias marinas, Tecnología y Gestión” (Do*MAR), Facultad de biología, Universidad de Vigo, Vigo, Spain;

^3^Centro Oceanográfico de Vigo (COV), Instituto Español de Oceanografía (IEO), CSIC. Subida a Radio Faro, 50. 36390 Vigo, Spain;

^4^CIBIO, Centro de Investigação em Biodiversidade e Recursos Genéticos, InBIO Laboratório Associado, Campus de Vairão, Universidade do Porto, 4485-661 Vairão, Portugal

^5^BIOPOLIS Program in Genomics, Biodiversity and Land Planning, CIBIO, Campus de Vairão, 4485-661 Vairão, Portugal

^6^Centro Oceanográfico de Cádiz (COCAD), Instituto Español de Oceanografía (IEO), CSIC, Puerto Pesquero, Muelle de Levante s/n, 11006 Cádiz, Spain;

^7^Tecnologías y Servicios Agrarios, S.A. (TRAGSATEC), C/ Orient, 63. 07760 Ciutadella, Spain;

^8^Department of Biology, Faculty of Sciences, University of Porto, Porto, Portugal.

^*^ Present address: Instituto de Investigaciones Marinas, Consejo Superior de Investigaciones Científicas (IIM-CSIC), Eduardo Cabello 6, 36208 Vigo, Spain

**+ These authors contributed equally to this work**

* **Corresponding authors:** L. Filipe C. Castro - filipe.castro@ciimar.up.pt; and Elsa Froufe – elsafroufe@gmail.com CIIMAR/CIMAR – Interdisciplinary Centre of Marine and Environmental Research, University of Porto, Terminal de Cruzeiros de Leixões, Matosinhos, Portugal.


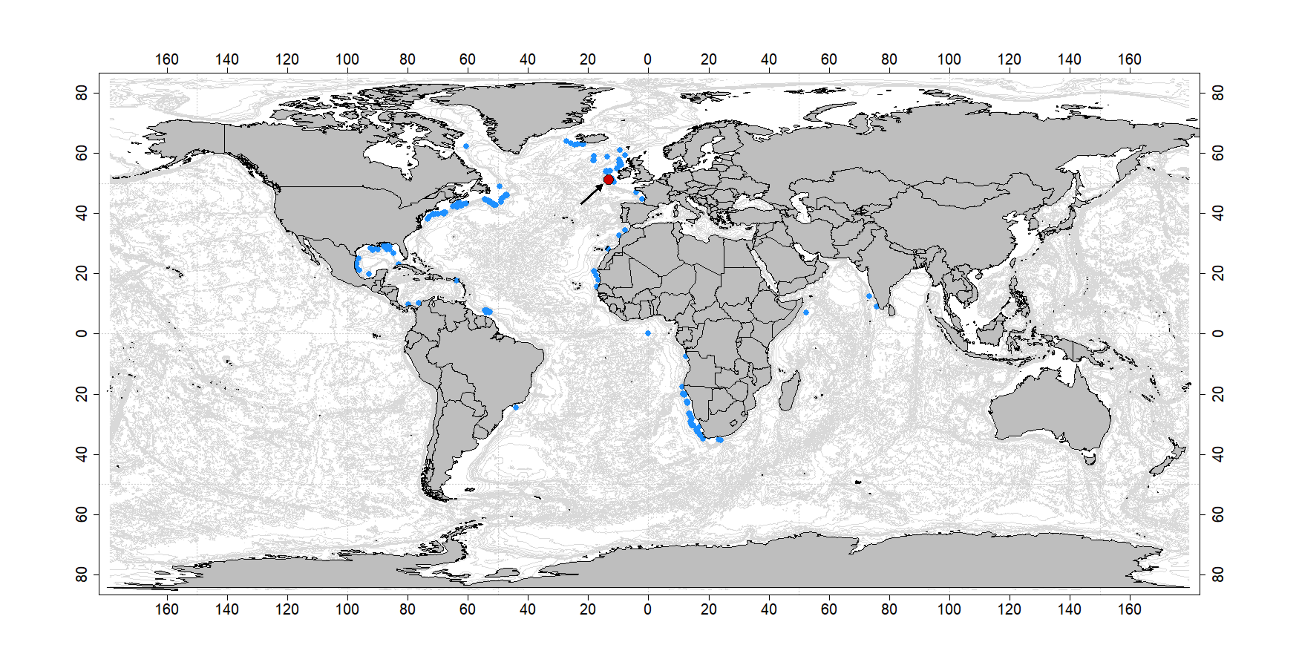
Supplementary Figure S1 - Distribution of Rhinochimaera atlantica. Geolocation data was downloaded from the Global Biodiversity Information Facility (GBIF) and Ocean Biogeographic Information System (OBIS) (date of access: 08/08/2023). The sampling location of the specimen studied is marked with an arrow.


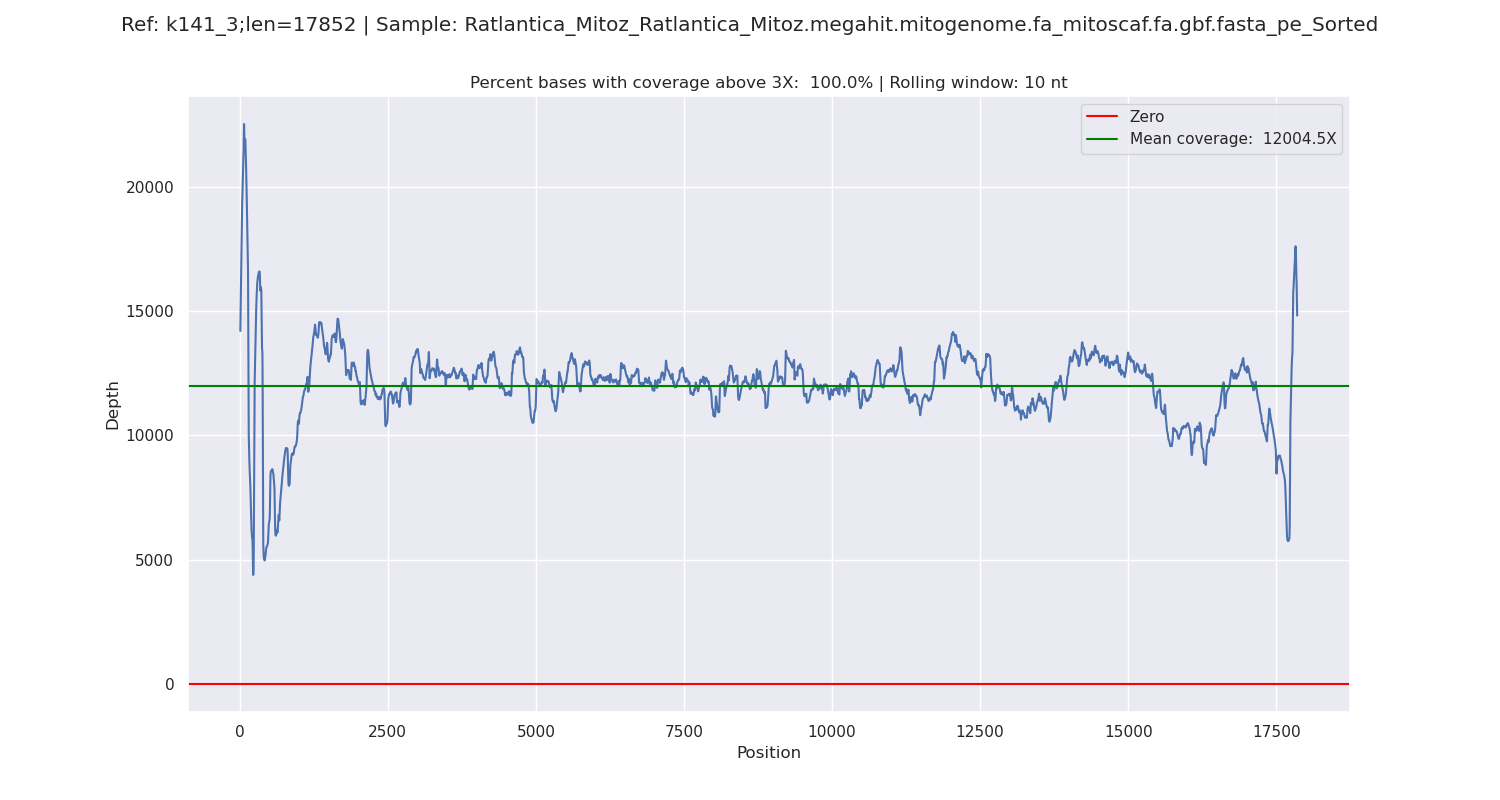


Supplementary Figure S2 - Read coverage plot of Rhinochimaera atlantica mitogenome.

Supplementary Table S1 – Detailed description of the male specimen *Rhinochimaera atlantica* (voucher name k141_3) morphometric measurements.

| **Species** | ***Rhinochimaera atlantica*** | | |
| --- | --- | --- | --- |
| **Measurement code** | **Acronym meanings** | **mm** |  |
| **TL** | Total length (TOT) | 1162 |  |
| **PCL** | Precaudal length | 863 |  |
| **BDL** | Body length | 491 |  |
| **SVL** | Snout-vent length | 638 |  |
| **TRL** | Trunk length | 254 |  |
| **PD2** | Pre-second dorsal length | 556 |  |
| **PD1** | Pre-first dorsal length | 407 |  |
| **POB** | Pre-orbital length | 301 |  |
| **POR** | Pre-oral length | 257 |  |
| **PRN** | Pre-narial length | 249 |  |
| **D2L** | Second dorsal length | 242 |  |
| **D2B** | Second dorsal fin base | 233 |  |
| **D2I** | Second dorsal inner margin | 10 |  |
| **D2AH =D2MH** | Maximum height of anterior second dorsal fin | 0 |  |
| **D2PH = D2MH** | Maximum height of posterior second dorsal fin | 0 |  |
| **D2H** | Maximum height of second dorsal fin | 30 |  |
| **D1B** | First dorsal fin base | 138 |  |
| **DSA** | Dorsal spine length | 96 |  |
| **D1H** | Maximum height of first dorsal fin | 79 |  |
| **CDM** | Dorsal caudal margin | 259 |  |
| **CDH =CUR** | Maximum height of dorsal caudal fin | 10 |  |
| **CTL** | Total caudal fin length including filament | 320 |  |
| **CVM** | Ventral caudal margin | 352 |  |
| **CVH =CLR** | Maximum height of ventral caudal fin | 40 |  |
| **HDL** | Head length | 384 |  |
| **P1A** | Anterior margin of pectoral fin | 215 |  |
| **P2A** | Anterior margin of pelvic fin | 111 |  |
| **IDS** | Interdorsal space | 21 |  |
| **DCS** | Dorsal-caudal space | 89 |  |
| **PPS** | Posterior base of pectoral fin to anterior base of pelvic fin | 220 |  |
| **PAS** | Posterior base of pelvic fin to origin of anal fin | 0 |  |
| **PCA** | Pelvic-caudal space | 144 |  |
| **PCS** | Posterior base of pelvic fin to origin of ventral caudal fin lobe | 0 |  |
| **D1P1** | Anterior edge of first dorsal fin base to anterior edge of pectoral fin base | 105 |  |
| **D1P2** | Anterior edge of first dorsal fin base to anterior edge of pelvic fin base | 260 |  |
| **D2P1** | Anterior edge of second dorsal fin base to anterior edge of pectoral fin base | 204 |  |
| **D2P2** | Anterior edge of second dorsal fin base to anterior edge of pelvic fin base | 147 |  |
| **EYL** | Eye length | 35 |  |
| **EYH** | Eye height | 19 |  |
| **EMO** | Eye-mouth space | 0 |  |
| **PP1** | Prepectoral length | 403 |  |
| **PP2** | Prepelvic length | 642 |  |
| **D1A** | First dorsal anterior margin | 128 |  |
| **D2MH = D2AH /D2PH** | Second dorsal midlength height | 0 |  |
| **CFI** | Caudal filament length | 25 |  |
| **CPW** | Caudal peduncle width | 14 |  |
| **GIR** | Girth | 294 |  |
| **P1L** | Pectoral length | 84 |  |
| **P2L** | Pelvic length | 63 |  |
| **P1W** | Pectoral fin width | 78 |  |
| **P2W** | Pelvic fin width | 47 |  |
| **P1B** | Pectoral fin base | 47 |  |
| **P2B** | Pelvic fin base | 28 |  |
| **P2P** | Space between the pectoral and pelvic fins | 0 |  |
| **HDH** | Head height | 109 |  |
| **HDW** | Head width | 65 |  |
| **TRH** | Trunk width | 135 |  |
| **TRW** | Trunk height | 89 |  |
| **TPH** | Trunk posterior height | 109 |  |
| **CPH** | Caudal-peduncle height | 30 |  |
| **MOL** | Mouth length | 12 |  |
| **MOW** | Mouth width | 29 |  |
| **LLA** | Lower labial furrow length | 21 |  |
| **ULA** | Upper labial furrow length | 19 |  |
| **ULH** | Upper labial fold height | 12 |  |
| **IOW** | Outer internarial width | 14 |  |
| **INO** | Interorbital space | 38 |  |
| **INW** | Internarial space | 22 |  |
| **ANF** | Anterior nasal flap legth | 5 |  |
| **NOW** | Nostril width | 4 |  |
| **GS1** | Gill opening height | 38 |  |
| **IG1** | Intergill widht | 36 |  |
| **SWF** | Snout greatest widht | 51 |  |
| **SWB** | Snout basal widht | 52 |  |
| **SHB** | Snout basal height | 48 |  |
| **AL** | Anal fin length | 0 |  |
| **CHI** | Maximum caudal heigth | 68 |  |
| **CUR=CDH** | Caudal upper ray length | 0 |  |
| **CLR =CVH** | Caudal lower ray length | 0 |  |
| **FTL** | Frontal tenaculum length | 27 |  |
| **FTB** | Frontal tenaculum base width | 7 |  |
| **FKW** | Frontal tenaculum knob width | 5 |  |
| **FKL** | Frontal tenaculum knob length | 10 |  |
| **PTL** | Prepelvic tenaculum length | 16 |  |
| **PTW** | Prepelvic tenaculum distal width | 16 |  |
| **PTB** | Prepelvic tenaculum base width | 6 |  |
| **CLT=CLO** | Total length of clasper | 91 |  |
| **CLM** | Length of medial branch of clasper (interior) | 0 |  |
| **CLL** | Length of lateral branch of clasper (exterior) | 0 |  |
| **CLB** | Clasper base width | 12 |  |
| **CLO =CLT** | Clasper outer length | 91 |  |
| **CLI** | Clasper inner length | 98 |  |
