# Supplementary figures and images for: The complete mitogenome of the Atlantic longnose chimaera Rhinochimaera atlantica (Holt & Byrne, 1909)
Source: Mitochondrial DNA B Resour. 2024 Jul 17;9(7):886–91. doi: 10.1080/23802359.2024.2378127 (PMC11257016; doi:10.1080/23802359.2024.2378127)

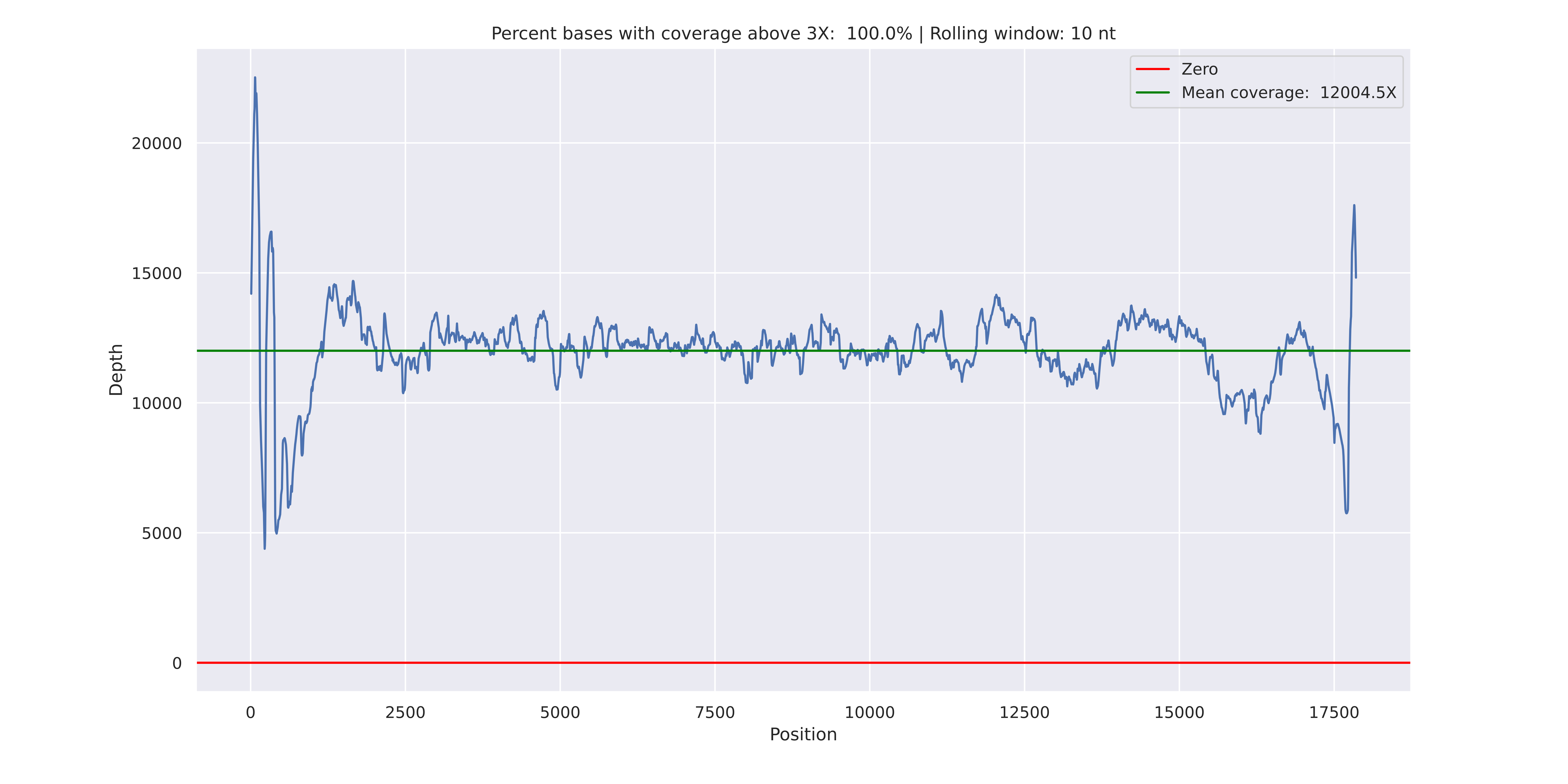

Supplement: Supplemental Material [file TMDN_A_2378127_SM0329.png]

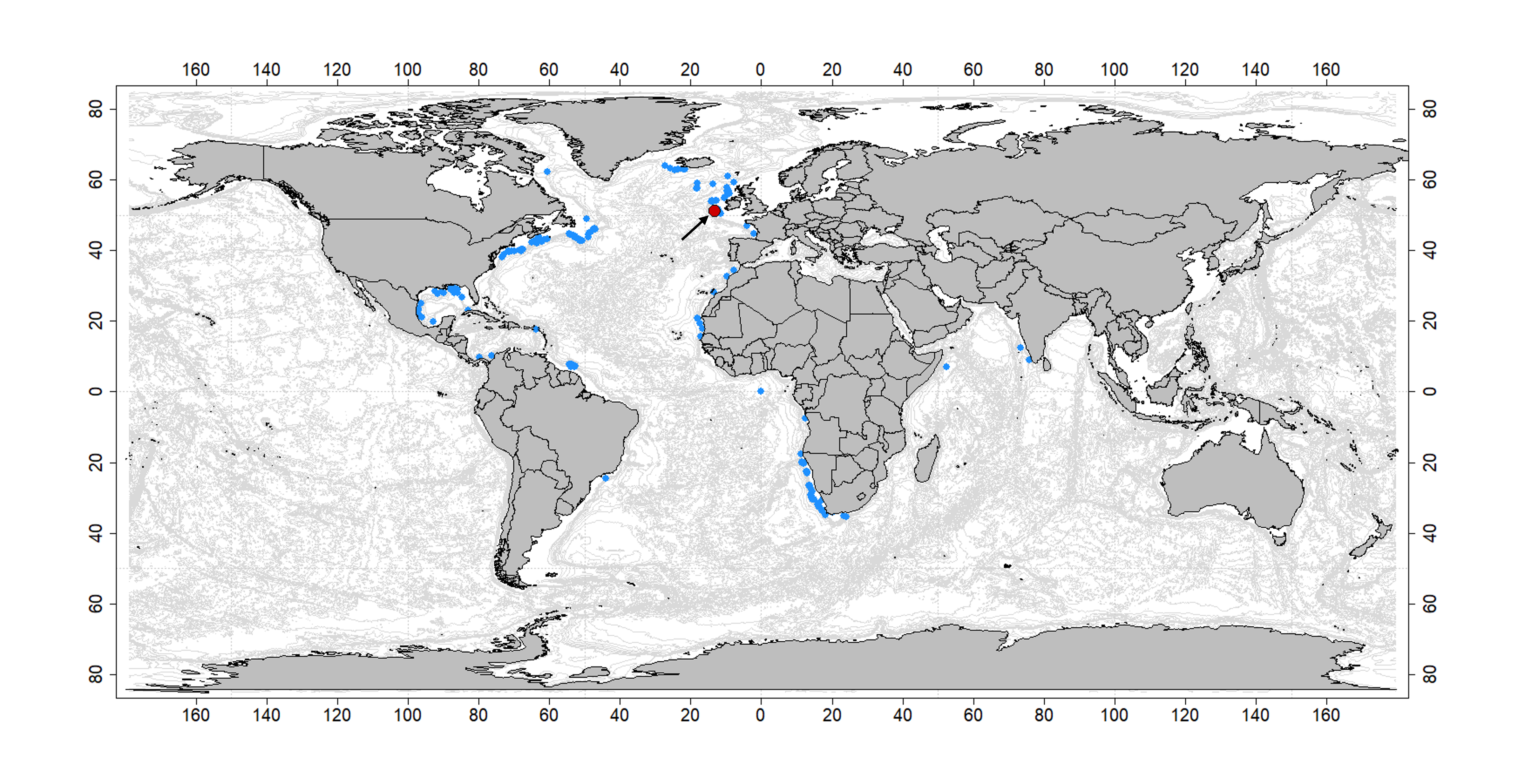

Supplement: Supplemental Material [file TMDN_A_2378127_SM0328.png]
